# Association Between Pelvic Asymmetry, Hip Joint Proprioception and Low Back Pain Severity in Patients with Lumbar Radiculopathy: A Cross-Sectional Comparative Study

**DOI:** 10.3390/jcm15218745

**Published:** 2026-11-09

**Authors:** Noura A. Hendy, Nahed A. Salem, Doaa A. Mekkawy, Hoda M. Zakaria, Zizi M. Ibrahim, Mohamed H. Marzouk

**Affiliations:** 1 Department of Physical Therapy for Neuromuscular Disorders and Its Surgery, Faculty of Physical Therapy, MTI University, Giza 11432, Egypt; staff@mc.mti.edu.eg; 2 Department of Physical Therapy for Neurology, Faculty of Physical Therapy, Cairo University, Giza 11432, Egypt; nahedahmed@cu.edu.eg (N.A.S.); dr.hodazakaria@cu.edu.eg (H.M.Z.); mohamedhelayel1990@cu.edu.eg (M.H.M.); 3 Department of Neurology, Faculty of Medicine, Cairo University, Giza 11432, Egypt; doaa.mekkawy@kasralainy.edu.eg; 4 Department of Rehabilitation Sciences, College of Health and Rehabilitation Sciences, Princess Nourah bint Abdulrahman University, P.O. Box 84428, Riyadh 11671, Saudi Arabia

**Keywords:** pelvic asymmetry, pelvic obliquity, pelvic tilt, hip joint proprioception, joint position error, lumbar radiculopathy, low back pain

## Abstract

**Background:** Lumbar radiculopathy (LR) is a clinical condition characterized by dysfunction of the lumbar nerve roots, often associated with nerve root compression, and commonly presents with low back pain (LBP). Pelvic asymmetry (PA) is characterized by frontal-plane pelvic obliquity (PO) and pelvic tilt asymmetry, defined as the side-to-side difference in the anterior superior iliac spine (ASIS)–posterior superior iliac spine (PSIS) angle in the sagittal plane. This asymmetry may be associated with alterations in lumbopelvic–hip biomechanics and mechanical load distribution across the lumbar spine, pelvis, and hip joints. Accurate proprioceptive feedback from the hip joint is essential for dynamic joint stability and coordinated lower-limb movement. However, the relationships among PA hip joint proprioception, and pain severity in patients with lumbar radiculopathy remain unclear. This study aimed to compare hip joint position error (JPE) and LBP intensity between patients with and without PA and to examine the correlations between pelvic alignment measures, hip joint JPE, and pain intensity. **Methods:** Sixty-four patients of either gender, aged 25–45 years, with LR due to lumbar disc herniation and presenting with LBP and unilateral radiating lower-extremity pain, were consecutively recruited. Participants were classified according to predefined cutoff values for PO and pelvic tilt asymmetry into two groups of 32: Group A (without PA) and Group B (with PA). Pelvic alignment was assessed using standardized photographs analyzed with Kinovea motion analysis software. Hip joint position sense was assessed using an isokinetic dynamometer, and LBP intensity was measured using the Numerical Pain Rating Scale (NPRS). **Results:** Patients in Group B demonstrated significantly greater JPE across all tested movements (flexion, abduction, and internal rotation) and higher pain intensity than patients in Group A (*p* < 0.05). Within Group B, pelvic obliquity was positively correlated with hip flexion, abduction, and internal rotation JPE (r = 0.47, 0.54, and 0.58, respectively; *p* < 0.05), but not with pain intensity (r = 0.30, *p* = 0.09). Pelvic tilt asymmetry was positively correlated with hip flexion JPE (r = 0.36), abduction JPE (r = 0.56), and pain intensity (r = 0.48) (all *p* < 0.05), but not with internal rotation JPE (r = 0.02, *p* = 0.91). **Conclusions**: Pelvic asymmetry is associated with greater hip joint position error, suggesting impaired hip joint position sense, and with higher pain intensity in patients with lumbar radiculopathy. However, the cross-sectional design precludes causal inferences. Longitudinal studies are warranted to further investigate these relationships.

## 1. Introduction

Lumbar radiculopathy (LR) is a painful disorder that arises when the lumbar nerve roots are compressed or irritated, most often at the L4-L5 and L5-S1 levels. Typical presentations include low back pain (LBP), shooting radicular pain, altered deep tendon reflexes, muscle weakness, numbness of the foot, and other sensory disturbances [1]. Lumbar disc herniation (LDH) is its leading cause, with a reported yearly incidence of 0.04 to 2.5 cases per 1000 people [2]. Roughly 95% of symptomatic herniations are found at the L4-L5 and L5-S1 levels [3].

The pelvis plays an important role in transferring and distributing mechanical loads between the trunk and lower extremities during standing and movement [4,5]. Within the lumbopelvic region, the sacrum provides a key connection between the vertebral column and the pelvic ring and therefore helps transmit forces between the trunk and lower limbs. Its position and orientation may be influenced by the magnitude and direction of loads transmitted through the spine and pelvis, as well as by the mechanical demands placed on the lumbopelvic region [6].

Pelvic asymmetry (PA) describes a condition in which the pelvic bones lose symmetrical alignment in one or more anatomical planes, including lateral tilting (obliquity) in the frontal plane, side-to-side differences in anterior–posterior iliac rotation in the sagittal plane, and axial rotation in the transverse plane. In the present study, sagittal-plane asymmetry was operationalized as the absolute side-to-side difference in the angle between the anterior superior iliac spine (ASIS) and posterior superior iliac spine (PSIS) on the two sides [7,8], whereas frontal-plane asymmetry was operationalized as pelvic obliquity (PO). Pelvic asymmetries may lead to uneven mechanical load distribution, which may increase stress on the lumbar soft tissues [9].

Pelvic obliquity (PO) is the angle formed between a horizontal reference line and the line joining the two ASIS in the frontal plane [10]. It develops when the pelvis fails to maintain horizontal alignment within the frontal plane. This uneven pelvic alignment may produce apparent hip asymmetry, with one iliac crest positioned higher than the other and has been linked to muscle imbalance, back pain, postural abnormalities, and gait disturbances [11]. Pelvic and spinal alignment can vary according to habitual postures and sitting positions, even in healthy individuals [12]. From a biomechanical perspective, differences in pelvic orientation may be accompanied by alterations in the lumbar–pelvic movement patterns [13]. Therefore, PA should be considered one of several interacting biomechanical factors that may be associated with LBP, rather than an independent causal factor.

Proprioception refers to the sensory information that the central nervous system receives from afferent receptors in peripheral muscles, joint capsules, ligaments, and joints. This information supports efficient neuromuscular control and provides the foundation for joint stability and postural balance, enabling precise awareness of absolute and relative body orientation [14]. As a key component of the motor control system, proprioception contributes to all stages of movement, including motor planning, execution, and correction [15]. It comprises joint position sense and the sense of joint movement (kinesthesia) [16]. Adequate afferent proprioceptive input from the periphery supports accurate joint position sense [17,18], whereas reduced afferent input may impair joint position perception and movement control [19]. Proprioceptive deficits are of particular interest in LBP, a major contributor to disability globally that places a heavy burden on economic and social development [20].

Altered pelvic tilt can change muscle length around the spine and hip, shifting spinal alignment and increasing stress on the lumbar vertebrae [21]. Previous studies have examined LBP in relation to sensorimotor function, pelvic alignment and PA; however, the specific relationship between PA and hip joint proprioception in patients with LR remains underexplored. Unlike nonspecific LBP, LR involves dysfunction of the lumbar nerve roots and is associated with different neurological manifestations [22]. These may affect sensorimotor function and movement control. Hip joint proprioception contributes to postural control and dynamic stability during gait [23]. In addition, PA has been associated with LBP [7], and altered lumbopelvic proprioception has been reported in selected populations with LBP. However, the relationships among PA, hip joint proprioception, and LBP severity in patients with LR remain insufficiently established.

Therefore, the aims of this study were (1) to compare hip joint position error (JPE) and LBP intensity between patients with LR with and without PA and (2) to examine the correlations of PO and pelvic tilt asymmetry with hip JPE and pain intensity. We hypothesized that patients with PA would show greater hip JPE and greater LBP intensity than those without PA. The findings may inform physiotherapy assessment and future research.

## 2. Materials and Methods

### 2.1. Study Design and Setting

This single-center cross-sectional comparative study was conducted in line with the Strengthening the Reporting of Observational Studies in Epidemiology (STROBE) guidelines for cross-sectional studies. Participants were allocated to one of two groups according to the presence or absence of PA. Between September 2025 and March 2026, we consecutively recruited 64 eligible patients who met the predefined inclusion and exclusion criteria from the outpatient clinic at the Modern University for Technology and Information (MTI). A neurologist established the diagnosis based on the clinical presentation and neurological examination, including LBP with radiating leg pain consistent with a radicular pattern and, when present, relevant neurological signs. Lumbar magnetic resonance imaging (MRI) confirmed disc herniation corresponding to the clinical presentation and the suspected affected nerve root.

### 2.2. Sample Size Calculation

An a priori power analysis was run in G*Power (version 3.0.10) to establish the required sample size. The inputs were a t-test family, linear multiple regression (fixed model, single regression coefficient), two-tailed testing, an alpha level of 0.05, a power of 0.8, and an effect size of 0.14 derived from an unpublished pilot study, with two independent groups assessed at a single time point. The analysis yielded a minimum total sample of 61 participants. Allowing for a 5% dropout rate, 64 participants (32 per group) were recruited.

### 2.3. Participants

A total of 64 participants of both genders, aged 25 to 45 years, were recruited for this study. All participants had chronic LR secondary to LDH, characterized by LBP with unilateral radiating lower extremity pain, with symptom duration ranging from 3 to 6 months. Participants were allocated to two groups according to the predefined cutoff value for PA and PO. The no PA Group (A) included 32 participants without PA, defined as PO ≤ 5.6° and pelvic tilt asymmetry ≤ 5°, whereas the PA Group (B) included 32 participants with PA, defined as PO > 5.6° and pelvic tilt asymmetry > 5°. Patients who met only one of the two criteria were excluded.

Patients were eligible if they: (1) were aged 25–45 years; (2) had chronic (3–6 months) LR with unilateral radiating leg pain secondary to MRI-confirmed LDH at L4–L5 or L5–S1; (3) had LBP; and (4) had the ability to stand unaided and to follow instructions. Exclusion criteria were previous spinal or lower-limb surgery; leg-length discrepancy, scoliosis, or other spinal deformity; hip, knee, or ankle pathology; sacroiliac joint dysfunction or pelvic fracture; bilateral radiculopathy, cauda equina syndrome, or progressive neurological deficit; systemic or neurological disorders affecting proprioception visual or vestibular disorders; and pregnancy.

**Figure 1:**
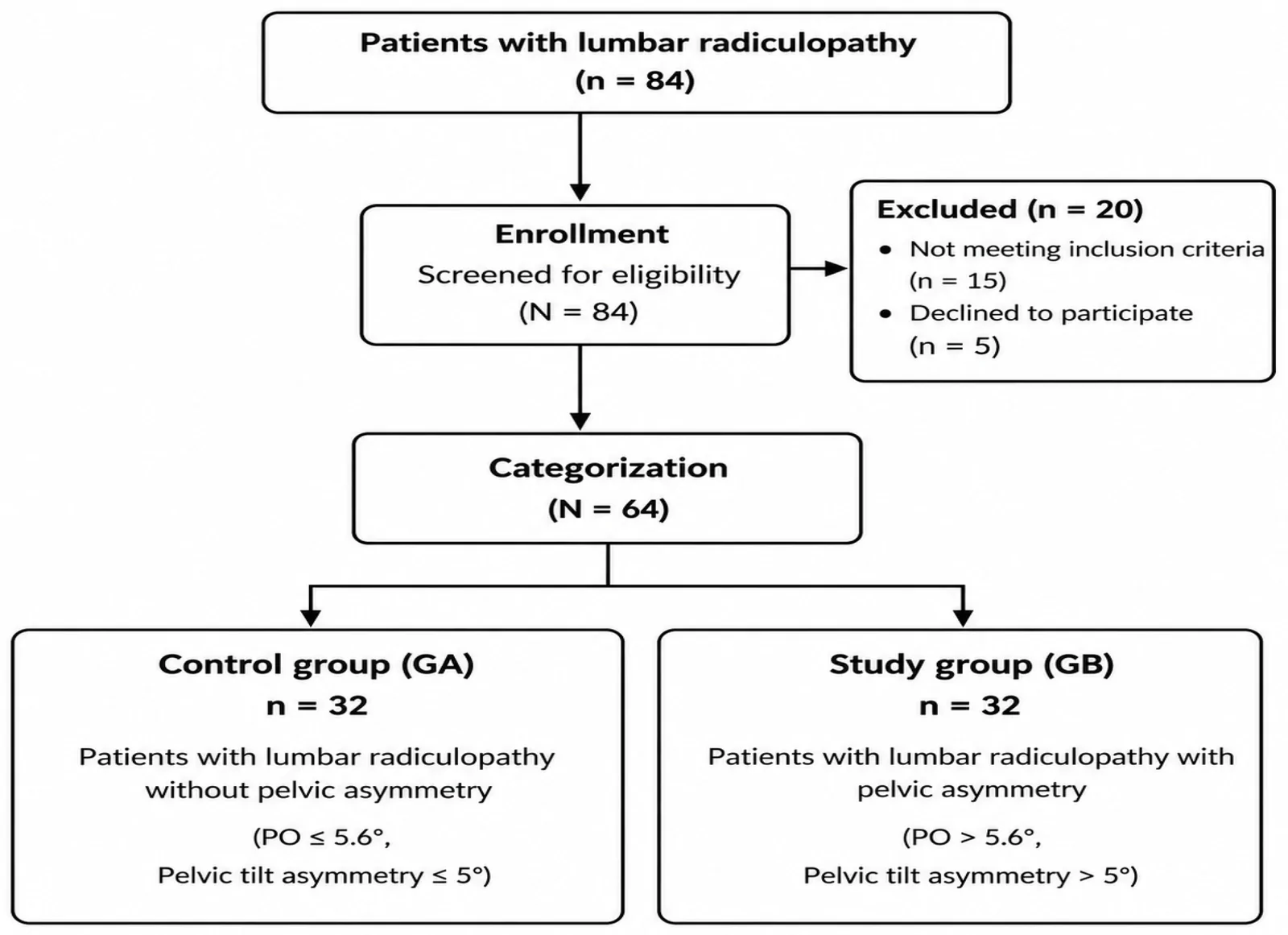
Participant Flow Diagram.

In the sagittal plane, pelvic tilt asymmetry represents the angular deviation between a level horizontal axis and the anatomical line linking the ASIS to the PSIS. It is typically evaluated in a neutral upright stance. Preece et al. reported good intratester reliability for the ASIS–PSIS measurement (intraclass correlation coefficient (ICC)= 0.92; standard error of measurement (SEM) = 0.5°). The left-right difference in ASIS–PSIS angles typically ranges from −6° to 5° [8]. In healthy individuals, PO typically ranges from 0° to 5.6°; a PO greater than 5.6° was therefore taken to indicate frontal-plane PA [24]. Figure 1 shows the participant flow.

### 2.4. Ethical Considerations

The study protocol was approved by the Ethical Committee of the Faculty of Physical Therapy, Cairo University (approval No. P.T.REC/012/006090, dated 2 September 2025), and the work followed the Declaration of Helsinki for research involving human participants. Before taking part, every participant was given detailed information about the aims, procedures, and potential requirements of the study, and written informed consent was obtained prior to enrollment. The study was observational, and no participant was assigned to a health-related intervention. All exposures were observed as part of routine clinical practice, and the study involved the retrospective analysis of existing clinical data. The study was voluntarily registered retrospectively on ClinicalTrials.gov (NCT07569510) on 8 May 2026, after data collection had been completed, to enhance transparency and public accessibility of the study information.

### 2.5. Assessment of Pelvic Asymmetry

#### 2.5.1. Taking Photos

Pelvic asymmetry was assessed using digital photographs of the anterior and lateral views of each participant’s pelvic region. The camera was mounted on a fixed stand, without rotation or tilt and positioned 1.5 m from the participant; the setup was calibrated using a standard non-elastic tape measurement. Camera height was also adjusted to match each patient’s pelvic height to ensure consistent perpendicular alignment [25]. Because the pelvic alignment measures were used to classify participants into the two groups, between-group differences in these measures follow from the classification criteria and are not independent findings. The main comparisons of interest were hip JPE and LBP intensity, which were not used to define the groups.

To standardize patient pelvic and lower limb posture, a self-balanced position was used. Patients performed active pelvic tilting movements into full anterior and posterior tilt to get into this posture. Patients then gradually decreased the range of motion until they stopped moving and kept the pelvis in a natural position. Participants were assessed in a relaxed standing position with the arms crossed over the chest, and the gaze fixed on a point on the wall directly in front of them and were asked to maintain a natural posture before photography. Plastic markers were taped bilaterally to the skin just above the ASIS and PSIS, and the photographs were then acquired.

#### 2.5.2. Measurement of the Pelvic Alignment Angles

Pelvic alignment angles were assessed from anterior and bilateral lateral photographs using Kinovea software. In the anterior view, a horizontal reference line was drawn through the right ASIS, and the angle between this line and the line joining the right and left ASIS was measured as PO. A PO > 5.6° indicated frontal-plane PA [24]. In the lateral views, the angle between a horizontal reference line through the ASIS and the line joining the ASIS and PSIS was measured on each side. A between-side difference >5° was taken to indicate sagittal-plane PA (pelvic tilt asymmetry). To improve measurement reliability, we obtained photographs under standardized conditions, with consistent participant positioning, anatomical landmark identification, camera placement, and measurement procedures. All measurements were performed using the same software and reference-line procedure. The assessor clearly marked each landmark before measurement and followed a standardized protocol to minimize measurement error.

### 2.6. Evaluation of Hip Joint Proprioception

Hip joint proprioception was evaluated with the Active Joint Position Sense (AJPS) test using a Biodex System 4 Quick-Set™ isokinetic dynamometer (Biodex Medical Systems Inc., Shirley, NY, USA). The test was performed during hip flexion, abduction, and internal rotation at an angular velocity of 30°/s. Participants were blindfolded throughout testing to eliminate visual feedback. The dynamometer axis was aligned with the anatomical axis of the hip, and standardized stabilization procedures were used to minimize compensatory movements.

For hip flexion and abduction, participants actively reproduced a target position of 25°, whereas a target of 20° was used for hip internal rotation. After reaching and memorizing the target position for 5 s, participants returned the limb to neutral (0°) and actively reproduced the target angle. The absolute difference between the target and reproduced angles (absolute error) was calculated by the Biodex software for each of the three trials, and the mean of the three trials was used as the JPE; lower values indicate better joint position sense.

#### Patient Preparation and Testing Procedures

Prior to testing, participants were given standardized verbal instructions and a demonstration of the required movements and wore an opaque eye mask to eliminate visual feedback. The dynamometer was adjusted according to the participant’s position, with its axis aligned with the anatomical axis of the hip and pelvis, and the tested limbs were firmly stabilized to minimize compensatory trunk and pelvic movements. All tests started from the neutral position (0°) and used an active-to-active joint position reproduction protocol at a constant angular velocity of 30°/s [26,27].

For hip flexion, participants were tested in the supine position with the pelvis stabilized and reproduced a target angle of 25°. For hip abduction, participants lay on the non-tested side with the pelvis stabilized and reproduced the 25° target angle [18]. For hip internal rotation, participants were seated with the hip and knee flexed to 90° and the trunk, pelvis, and tested thigh stabilized; the target angle was 20° [28]. For each movement, participants actively reached the target, held it for 5 s to memorize the position, returned to 0°, and then actively reproduced it. The reproduced position was recorded when the participant indicated that the target had been reached.

Several procedures were implemented to enhance reliability: standardized participant positioning, consistent dynamometer alignment and stabilization, fixed target angles, a constant angular velocity, standardized instructions, and the same testing sequence for all participants. Three repeated trials were performed for each movement, and their mean was used to reduce the influence of random trial-to-trial variation [26,27].

### 2.7. Assessment of Low Back Pain Intensity

Low back pain intensity was assessed with the Numerical Pain Rating Scale (NPRS, a tool used widely in both clinical practice and research, including studies of people with low back pain [29]. Participants rated their average low back pain over the past 24 h on an 11-point scale, where 0 meant no pain, and 10 meant the worst pain imaginable [30]. Each participant chose one whole number that best matched how intense their pain was, and this score was entered into the statistical analysis. Pain intensity was classified as none (0), mild (1–3), moderate (4–6), or severe (7–10) [31,32,33].

### 2.8. Statistical Analysis

All analyses were carried out in IBM SPSS Statistics (version 23; IBM Corp., Chicago, IL, USA), with a two-tailed significance level of α = 0.05. Before testing, the data were screened for normality, homogeneity of variance, and extreme values, and the Shapiro–Wilk test showed that all variables followed a normal distribution (*p* > 0.05). Continuous variables are presented as mean ± standard deviation (SD), while categorical variables (sex, affected side, and affected level) are reported as frequencies and percentages. Group differences in baseline characteristics were tested with independent-samples t-tests for continuous variables and chi-square tests for categorical variables. To compare the outcome variables between groups, a one-way multivariate analysis of variance (MANOVA) was used. Before running this analysis, its underlying assumptions were verified, namely multivariate normality, linearity, absence of multicollinearity, and homogeneity of variance–covariance matrices.

When the MANOVA was significant, follow-up univariate analyses of variance (ANOVAs) with Bonferroni correction were performed to control for Type I error. The relationships between pelvic alignment variables and JPE/pain intensity were evaluated using Pearson’s correlation coefficients and their 95% confidence intervals (CIs).

## 3. Results

### 3.1. Demographic Characteristics of Participants

As shown in Table 1, baseline demographic characteristics were comparable between the two groups (all *p* > 0.05).

### 3.2. Results of the One-Way MANOVA

A one-way MANOVA was performed to test whether the two groups differed across the outcome variables. The overall multivariate effect of group was statistically significant (Wilks’ Λ = 0.13, F(6, 57) = 62.99, *p* < 0.001, partial η^2^ = 0.87).

#### Between-Group’s Analysis

The results demonstrated statistically significant differences between the two groups in hip flexion JPE, hip abduction JPE, hip internal rotation JPE, PO angle, pelvic tilt asymmetry, and NPRS scores. As expected, based on the group classification criteria, Group B (with PA) demonstrated greater PO and pelvic tilt asymmetry. More importantly, Group B also showed greater JPE in all three movements and higher pain intensity than Group A (without PA), as shown in Table 2.

### 3.3. Correlation Between Pelvic Asymmetry, Hip Joint Proprioception and NPRS

Within the PA group, pelvic obliquity demonstrated significant, moderate, positive correlations with hip flexion, abduction, and internal rotation JPE (r = 0.47–0.58), indicating that greater pelvic obliquity was associated with greater hip joint position error. In contrast, the correlation with pain intensity was weak and non-significant (r = 0.30, *p* = 0.09). Pelvic tilt asymmetry was significantly and positively correlated with hip flexion JPE (r = 0.36), hip abduction JPE (r = 0.56), and pain intensity (r = 0.48), but it was not correlated with hip internal rotation JPE (r = 0.02). These correlations reflect associations within the PA group and should not be interpreted as evidence of a causal relationship. Correlation coefficients and *p* values are given in Table 3.

## 4. Discussion

This study investigated the associations among PA, hip joint proprioception, and LBP severity in patients with LR. The principal findings were that patients with PA (Group B: PO > 5.6° and pelvic tilt asymmetry > 5°) had greater hip JPE in flexion, abduction, and internal rotation and higher pain intensity than patients without PA (Group A: PO ≤ 5.6° and pelvic tilt asymmetry ≤ 5°), and that, within Group B, PO and pelvic tilt asymmetry were differentially correlated with JPE and pain intensity.

Although the between-group differences were statistically significant, their magnitude should be interpreted against the measurement error of the assessment procedures. The mean differences ranged from 1.49° (hip internal rotation JPE) to 4.04° (hip abduction JPE). The smallest difference, for hip internal rotation JPE (1.49°; 95% CI, 1.12–1.87°), should be interpreted in light of the measurement error of the Biodex AJPS test. Previous studies have reported acceptable reliability of Biodex-based joint position sense measurements [26,27]; however, reliability was not established in the present sample. Statistical significance alone does not establish clinical importance; differences that clearly exceed the measurement error provide stronger evidence of true differences rather than random variability.

The findings indicate that PA is associated with greater hip JPE, suggesting poorer hip joint position sense, and with higher pain intensity in Group B than in Group A. This is consistent with Hillier et al. [34], who reported impaired proprioception in various musculoskeletal pain conditions, particularly chronic LBP.

Pelvic asymmetry may disrupt sacroiliac joint biomechanics and may alter hip joint proprioception by changing pelvic alignment and redistributing mechanical loads across the hip and surrounding muscles. These changes may affect sensorimotor control and contribute to altered muscle activation patterns, potentially influencing lumbopelvic stability and LBP [35]. However, the relationship may be bidirectional, because impaired proprioception and altered neuromuscular control may also contribute to abnormal pelvic positioning. Reverse causation is also possible for pain: antalgic postures adopted by patients with disc herniation (e.g., lateral shift) may themselves produce pelvic obliquity.

These biomechanical changes may induce protective shifts in muscle activation patterns and alter mechanoreceptor input, compromising sensorimotor control and reducing joint position sense accuracy [36,37]. Korakakis et al. [38] also reported impaired lumbopelvic proprioception in subgroups of individuals with non-specific LBP, which supports the presence of proprioceptive deficits in lumbopelvic disorders.

However, Shoukry et al. [39] reported that anterior innominate rotation, a form of PA, did not significantly affect weight-bearing joint position sense. One possible explanation is that weight-bearing assessments draw on sensory information from multiple joints and surrounding structures, which may compensate for or mask subtle proprioceptive deficits. In addition, detecting small differences in the relative rotation of the innominate bones is challenging, and the precision and reliability of the assessment methods may limit such measurements. In contrast, the present study examined PO and pelvic tilt asymmetry and assessed hip proprioception in non-weight-bearing positions (supine, side-lying, and seated). This protocol minimized compensatory sensory input from the knee and ankle joints, which may explain the discrepancy between the two studies’ findings.

The current findings revealed significant correlations between PO and hip JPE in all tested movements. This is consistent with Zhou et al. [40], who reported that PO influences acetabular orientation, joint congruency, and load distribution; these biomechanical alterations may affect sensorimotor control and contribute to the proprioceptive deficits observed here.

From a biomechanical perspective, PO may alter hip joint mechanics and the activity of the surrounding muscles, particularly the hip abductors, which contribute to pelvic stability during functional movements [41]. Changes in muscle length and activation may alter proprioceptive input, potentially affecting hip joint position sense. These alterations may also affect lumbopelvic control and contribute to LBP [42]. Proprioception relies on afferent input from receptors in muscles, joints, and surrounding tissues. Therefore, changes in pelvic alignment and associated changes in hip mechanics may disrupt sensory feedback and thereby contribute to increased JPE [37].

The current study found that pelvic tilt asymmetry was significantly associated with greater JPE during hip flexion and abduction. This aligns with Donati et al. [43], who reported that sagittal-plane deviations alter hip biomechanics, including acetabular orientation and range of motion. Alterations in pelvic tilt may modify hip joint loading, muscle forces, and movement mechanics [44,45], which could impair proprioceptive accuracy by changing sensory input from joint and muscular mechanoreceptors.

Kuhns et al. [46] reported in a systematic review that changes in sagittal pelvic alignment affect hip range of motion and joint mechanics. Such biomechanical changes may modify mechanoreceptor stimulation around the hip joint and thereby reduce proprioceptive acuity. This aligns with Gu et al. [47], who reported significant improvements in joint position sense after feedback-based pelvic tilt training in patients with chronic stroke, suggesting that optimizing pelvic control and mechanics may improve proprioceptive accuracy.

In contrast, Jung et al. [48] observed that improvements in pelvic tilting angle were not accompanied by significant changes in proprioceptive accuracy among individuals with hamstring tightness. This discrepancy suggests that changes in pelvic mechanics do not always translate into measurable improvements in proprioceptive function, particularly when muscular tightness limits movement.

However, we found no significant relationship between pelvic tilt asymmetry and hip internal rotation JPE. This is consistent with Neumann [42], who suggested that hip internal rotation is influenced primarily by local joint structures, including the ischiofemoral ligament, rather than global lumbopelvic alignment. This interpretation is further supported by Alkhamis et al. [49], who showed that structural alterations within the hip joint were associated with impaired proprioceptive function, suggesting that local joint structures and mechanoreceptors play a critical role in maintaining proprioceptive accuracy.

This finding may also be explained by the seated assessment position (90° hip flexion) used for this movement. Endo et al. [50] reported that sitting increases posterior pelvic tilt and reduces lumbar lordosis, making sagittal pelvic alignment more position-dependent than during standing. Although sagittal pelvic tilt can influence femoral rotational kinematics during weight-bearing activities [51], proprioceptive input during seated non-weight-bearing internal rotation is largely mediated by local mechanoreceptors and surrounding musculature [52,53]. Conversely, PO showed a significant relationship with JPE for hip internal rotation. Unlike sagittal alignment, frontal-plane asymmetry may persist in sitting, potentially allowing it to continue influencing hip joint alignment and proprioceptive accuracy. This explanation is speculative because pelvic alignment was not measured in the seated position.

The present study found a weak, non-significant positive correlation between PO and pain intensity. This aligns with In et al. [54], who studied sitting posture in adolescents with LBP and found no significant differences in frontal-plane PA between those with and without pain, suggesting that PO alone may not adequately explain pain severity or occurrence.

This finding may reflect the multifactorial nature of LBP, as multiple biological, psychological, and social factors influence pain intensity. Furthermore, PA is frequently observed in asymptomatic individuals, suggesting that asymmetry alone may not be sufficient to provoke pain [8,24,55]. In contrast, pelvic tilt asymmetry demonstrated a significant relationship with pain intensity. This is consistent with Yu et al. [7], who reported that sagittal-plane asymmetry was significantly associated with chronic non-specific LBP, whereas frontal-plane asymmetry showed no significant relationship.

### 4.1. Strengths & Limitations

The present study has several notable strengths. It addressed a relatively underexplored area in patients with LR by examining the association between PA and hip joint proprioception, providing further insight into the biomechanical and sensorimotor mechanisms that may contribute to movement impairment in this population. In addition, the study used objective, quantitative assessment techniques, including digital motion analysis to evaluate pelvic alignment and an isokinetic dynamometer to assess hip joint position sense, which improved the objectivity of the measurements. Another strength was the use of clearly defined criteria for identifying pelvic asymmetry, allowing a more focused comparison between individuals with and without PA and facilitating a better understanding of its potential impact on proprioceptive function and pain severity. Furthermore, the inclusion of participants of both genders broadens the applicability of the findings, and the similarity of baseline characteristics between the groups supports their comparability.

Despite the strengths of this study, some limitations should be considered when interpreting the findings. First, the cross-sectional design does not allow us to determine whether PA contributes to impaired proprioception and increased pain, or whether these findings are related to the underlying condition; causality cannot be established. Future longitudinal and interventional studies are needed to better understand these relationships and to investigate whether interventions targeting pelvic alignment can improve proprioception and clinical outcomes. Second, pelvic alignment was assessed using photographs and surface landmarks identified at the ASIS and PSIS rather than direct three-dimensional measurements of the iliac bones. Differences in landmark identification, participant positioning, and the limitations of the photographic method may have affected the measurements. Therefore, the results should be interpreted as differences in the measured pelvic alignment angles rather than direct evidence of underlying iliac bone rotation. Third, the study included only individuals aged 25–45 years with l LR secondary to lumbar disc herniation. As a result, the findings may not apply to other age groups or to patients with LBP caused by other conditions. In addition, although objective assessment tools were used, we did not assess other factors that may influence proprioception and pain, including physical activity levels, psychological factors, and habitual postural behaviors. The study also assessed LBP intensity using the NPRS but did not measure disability or radiating leg pain. Including these outcomes in future research would help provide a more complete picture of the effects of LR on patients’ symptoms and daily activities. The study was also conducted at a single center with a modest sample size and without a healthy control group. Finally, the study focused mainly on pelvic alignment and hip joint proprioception. Future studies that include a broader range of sensorimotor, functional, and clinical outcomes may help clarify how PA relates to movement control and its potential effects on patients’ daily functioning.

### 4.2. Clinical Implications

The findings of this study may provide preliminary insight into the relationship between PA and hip joint proprioception in patients with LBP. Evaluating PA alongside hip joint proprioception may provide complementary information about these patients’ musculoskeletal and sensorimotor characteristics. However, clinicians should not assume PA causes proprioceptive impairment or LBP based on an observed association alone. Further studies are needed to establish the clinical relevance and measurement feasibility of these assessments and to determine whether interventions addressing these variables can meaningfully improve pain, functional performance, and rehabilitation outcomes in this population.

## 5. Conclusions

In this cross-sectional comparative study, patients with LR due to LDH who were classified as having PA showed greater hip JPE and higher LBP intensity than those without PA. Within the pelvic-asymmetry group, pelvic alignment measures were associated differently with hip JPE and pain intensity. Overall, the findings suggest an association among pelvic alignment, hip joint position sense, and LBP intensity. However, because the study was cross-sectional, no cause-and-effect relationship can be established, and longitudinal studies are needed to determine the clinical importance of these findings.

## Figures and Tables

**Table 1:** Baseline demographic characteristics of participants (N = 64).

Characteristics	Group A (Without Pelvic Asymmetry)	Group B (with Pelvic Asymmetry)	Test Statistic (t or χ^2^)	*p* Value
**Age (years)**	38.28 ± 6.03	39.5 ± 5.36	−0.85	0.4
**Weight (kg)**	62 ± 7.83	64.69 ± 6.79	−1.47	0.15
**Height (cm)**	165.28 ± 8.2	166.75 ± 8.18	−0.72	0.48
**BMI (kg/m^2^)**	22.61 ± 1.39	23.23 ± 1.4	−1.78	0.08
**Chronicity of lumbar radiculopathy (weeks)**	17.81 ± 2.69	18.53 ± 2.82	−1.04	0.3
**Gender, n (%)**				
**Female**	18 (56.25%)	22 (68.75%)	χ^2^ = 1.07	0.3
**Male**	14 (43.75%)	10 (31.25%)		
**Affected side, n (%)**				
**Right**	15 (46.88%)	19 (59.38%)	χ^2^ = 1	0.32
**Left**	17 (53.12%)	13 (40.62%)		
**Affected level, n (%)**				
**L4/5**	21 (65.63%)	18 (56.25%)	χ^2^ = 0.59	0.44
**L5/S1**	11 (34.37%)	14 (43.75%)		

BMI, body mass index; χ^2^, chi-square test; SD, standard deviation. Data are mean ± SD, except for sex, affected side, and affected level, which are presented as n (%). *p* > 0.05 indicates no statistically significant difference.

**Table 2:** Comparison of the outcome measures between the two groups (N = 64).

Outcome Measures	Group A (Without Pelvic Asymmetry)	Group B (with Pelvic Asymmetry)	MD (95%CI)	*p*-Value	Sign
	$\bar{Χ}$ ± SD	$\bar{Χ}$ ± SD			
**Hip flexion JPE (degree)**	4.73 ± 1.33	7.51 ± 1.13	−2.78 (−3.39, −2.16)	<0.001	S
**Hip abduction JPE (degree)**	3.43 ± 0.4	7.47 ± 1.16	−4.04 (−4.66, −3.41)	<0.001	S
**Hip internal rotation JPE (degree)**	2.45 ± 0.74	3.94 ± 0.77	−1.49 (−1.87, −1.12)	<0.001	S
**Pelvic obliquity angle (degree)**	2.9 ± 0.94	6.31 ± 0.5	−3.41 (−3.88, −2.94)	<0.001	S
**Pelvic tilt asymmetry (degree)**	2.95 ± 1.01	6.76 ± 0.92	−3.81 (−4.29, −3.33)	<0.001	S
**NPRS (cm)**	4.28 ± 1.05	7.06 ± 1.5	−2.78 (−3.68, −1.88)	<0.001	S

Data are mean ± SD. MD, mean difference (Group A − Group B; negative values indicate higher values in Group B); CI, confidence interval; JPE, joint position error; NPRS, Numerical Pain Rating Scale. *p*-values are from univariate ANOVAs with Bonferroni correction; S, statistically significant (*p* < 0.05).

**Table 3:** Correlation between pelvic alignment measures, hip joint position error and pain intensity.

Pelvic Alignment Variables	Hip Proprioception and Pain Variables	r Value	*p* Value	Sig
**Pelvic obliquity angle (degree)**	Hip flexion JPE **(degree)**	0.47	0.007	**S**
	Hip abduction JPE **(degree)**	0.54	0.002	**S**
	Hip internal rotation JPE **(degree)**	0.58	0.001	**S**
	NPRS (cm)	0.3	0.09	**NS**
**Pelvic tilt asymmetry**	Hip flexion JPE **(degree)**	0.36	0.046	**S**
	Hip abduction JPE **(degree)**	0.56	0.001	**S**
	Hip internal rotation JPE **(degree)**	0.02	0.91	**NS**
	NPRS (cm)	0.48	0.004	**S**

r, Pearson correlation coefficient; *p*, probability value; S, statistically significant; NS, not statistically significant; JPE, joint position error; NPRS, Numerical Pain Rating Scale; cm, centimeters.

## Data Availability

The data supporting this study’s findings are available on reasonable request.

## Abbreviations

The following abbreviations are used in this manuscript:

A JPS	Active Joint Position Sense
ANOVA	Analysis of Variance
ASIS	Anterior Superior Iliac Spine
BMI	Body Mass Index
JPE	Joint Position Error
LBP	Low Back Pain
LDH	Lumbar Disc Herniation
LR	Lumbar Radiculopathy
MANOVA	Multivariate Analysis of Variance
MD	Mean Difference
MRI	Magnetic Resonance Imaging
NPRS	Numerical Pain Rating Scale
PA	Pelvic Asymmetry
PO	Pelvic Obliquity
PSIS	Posterior Superior Iliac Spine

## References

[1] Vanti C., Panizzolo A., Turone L., Guccione A.A., Violante F.S., Pillastrini P., Bertozzi L. (2021). Effectiveness of mechanical traction for lumbar radiculopathy: A systematic review and meta-analysis. Phys. Ther..

[2] Brauer C., Mikkelsen S., Pedersen E.B., Møller K.L., Simonsen E.B., Koblauch H., Thygesen L.C. (2020). Occupational lifting predicts hospital admission due to low back pain in a cohort of airport baggage handlers. Int. Arch. Occup. Environ. Health.

[3] Hincapié C.A., Kroismayr D., Hofstetter L., Kurmann A., Cancelliere C., Raja Rampersaud Y., Cassidy J.D. (2025). Incidence of and risk factors for lumbar disc herniation with radiculopathy in adults: A systematic review. Eur. Spine J..

[4] Kowal M., Starczewska A., Rutkowska-Kucharska A. (2018). Biomechanical parameters of gait after unilateral above-knee amputation. Current state of research. Ortop. Traumatol. Rehabil..

[5] Bibrowicz K., Szurmik T., Ogrodzka-Ciechanowicz K., Hudakova Z., Gąsienica-Walczak B., Kurzeja P. (2023). Asymmetry of the pelvis in Polish young adults. Front. Psychol..

[6] Ren B.O., Goldberg R.W., Standefer K.D., Teplensky J.R., Drain J.P., McCarthy C.F., Liu R. (2024). Analyzing pelvic asymmetry by sex and ancestry: Insights from an osteological collection. Cureus.

[7] Yu Q., Huang H., Zhang Z., Hu X., Li W., Li L., Chen M., Liang Z., Lo W.L.A., Wang C. (2020). The association between pelvic asymmetry and non-specific chronic low back pain as assessed by the Global Postural System. BMC Musculoskelet. Disord..

[8] Preece S.J., Willan P., Nester C.J., Graham-Smith P., Herrington L., Bowker P. (2008). Variation in pelvic morphology may prevent the identification of anterior pelvic tilt. J. Man. Manip. Ther..

[9] Bibrowicz K., Ogrodzka-Ciechanowicz K., Hudakova Z., Szurmik T., Bibrowicz B., Kurzeja P. (2025). Pelvic asymmetry and stiffness of the muscles stabilizing the lumbo–pelvic–hip complex (LPHC) in tensiomyography examination. J. Clin. Med..

[10] Saetang L., Sakulsriprasert P., Vongsirinavarat M. (2015). The adaptive patterns of pelvic alignment in individuals with adolescent idiopathic scoliosis. J. Phys. Ther. Sci..

[11] Hägglund G. (2020). Association between pelvic obliquity and scoliosis, hip displacement and asymmetric hip abduction in children with cerebral palsy: A cross-sectional registry study. BMC Musculoskelet. Disord..

[12] Liu Y., Zhang Y.H., Lim H.W. (2025). Effects of smartphone use in various floor sitting positions on spinal and pelvic alignment and gluteal pressure in healthy adults. J. Phys. Ther. Sci..

[13] Nishimura T., Tanaka M., Morikoshi N., Yoshizawa T., Miyachi R. (2025). Differences in lumbar–pelvic rhythm between sedentary office workers with and without low back pain: A cross-sectional study. Healthcare.

[14] Cheng X., Yang J., Hao Z., Li Y., Fu R., Zu Y., Wang C. (2023). The effects of proprioceptive weighting changes on posture control in patients with chronic low back pain: A cross-sectional study. Front. Neurol..

[15] Héroux M.E., Butler A.A., Robertson L.S., Fisher G., Gandevia S.C. (2022). Proprioception: A new look at an old concept. J. Appl. Physiol..

[16] Alahmari K.A., Reddy R.S., Silvian P.S., Ahmad I., Kakaraparthi V.N., Alam M.M. (2017). Association of age on cervical joint position error. J. Adv. Res..

[17] Alshahrani M.S., Reddy R.S., Tedla J.S., Asiri F., Alshahrani A. (2022). Association between kinesiophobia and knee pain intensity, joint position sense, and functional performance in individuals with bilateral knee osteoarthritis. Healthcare.

[18] Asiri F., Reddy R.S., Narapureddy B.R., Raizah A. (2022). Comparisons and associations between hip-joint position sense and glycosylated hemoglobin in elderly subjects with Type 2 Diabetes Mellitus: A cross-sectional study. Int. J. Environ. Res. Public Health.

[19] Labanca L., Ciardulli F., Bonsanto F., Sommella N., Di Martino A., Benedetti M.G. (2021). Balance and proprioception impairment, assessment tools, and rehabilitation training in patients with total hip arthroplasty: A systematic review. BMC Musculoskelet. Disord..

[20] GBD 2021 Low Back Pain Collaborators (2023). Global, regional, and national burden of low back pain, 1990–2020, its attributable risk factors, and projections to 2050: A systematic analysis of the Global Burden of Disease Study 2021. Lancet Rheumatol..

[21] Kim W.D., Shin D. (2023). Effects of pelvic-tilt imbalance on disability, muscle performance, and range of motion in office workers with non-specific low-back pain. Healthcare.

[22] Berry J.A., Elia C., Saini H.S., Miulli D.E. (2019). A review of lumbar radiculopathy, diagnosis, and treatment. Cureus.

[23] Qu X., Hu X., Zhao J., Zhao Z. (2022). The roles of lower-limb joint proprioception in postural control during gait. Appl. Ergon..

[24] Moharrami A., Mirghaderi P., Hoseini Zare N., Moazen-Jamshidi M.M., Ebrahimian M., Mortazavi S.M.J. (2023). Slight pelvic obliquity is normal in a healthy population: A cross-sectional study. J. Exp. Orthop..

[25] Hazar Z., Karabicak G.O., Tiftikci U. (2015). Reliability of photographic posture analysis of adolescents. J. Phys. Ther. Sci..

[26] Matsui T., Hirata K., Miyamoto N., Akagi R. (2025). Adaptive walking performance is related to the hip joint position sense during active hip flexion rather than during passive hip flexion. Front. Sports Act. Living.

[27] Katsaveli I., Kellari A., Dimitriadis Z., Poulis I., Kanellopoulos A. (2023). Investigation of the knee angular velocity proprioceptive behavior as the joint velocity increases. Gait Posture.

[28] Onishi H., Nagoya S., Takebayashi T., Yamashita T. (2017). Analysis of proprioception of hip joint in total hip arthroplasty. Open J. Orthop..

[29] Chiarotto A., Maxwell L.J., Ostelo R., Boers M., Tugwell P., Terwee C. (2019). Measurement Properties of Visual Analogue Scale, Numeric Rating Scale, and Pain Severity Subscale of the Brief Pain Inventory in Patients With Low Back Pain: A Systematic Review. J. Pain. Off. J. Am. Pain. Soc..

[30] Goudman L., Pilitsis J., Billet B., De Vos R.D., Hanssens K., Billot M., Roulaud M., Rigoard P., Moens M. (2023). The level of agreement between the numerical rating scale and visual analogue scale for assessing pain intensity in adults with chronic pain. Anaesthesia.

[31] Turk D.C., Melzack R. (2011). Handbook of Pain Assessment.

[32] Shafshak T., Elnemr R. (2020). The Visual Analogue Scale Versus Numerical Rating Scale in Measuring Pain Severity and Predicting Disability in Low Back Pain. JCR J. Clin. Rheumatol..

[33] Williamson A., Hoggart B. (2005). Pain: A review of three commonly used pain rating scales. J. Clin. Nurs..

[34] Hillier S., Immink M., Thewlis D. (2015). Assessing proprioception: A systematic review of possibilities. Neurorehabil. Neural Repair..

[35] Cruz-Medel I., Rodrigues-de-Souza D.P., Alburquerque-Sendín F. (2024). Comprehensive analysis of pelvic asymmetries in low back pain, scoliosis, post-traumatic pelvic dysfunctions and obstetric changes: A narrative review focused on clinical relevance. Symmetry.

[36] Hodges P.W., Tucker K. (2011). Moving differently in pain: A new theory to explain the adaptation to pain. Pain.

[37] Proske U., Gandevia S.C. (2012). The proprioceptive senses: Their roles in signaling body shape, body position and movement, and muscle force. Physiol. Rev..

[38] Korakakis V., O’Sullivan K., Kotsifaki A., Sotiralis Y., Giakas G. (2021). Lumbo-pelvic proprioception in sitting is impaired in subgroups of low back pain–But the clinical utility of the differences is unclear. A systematic review and meta-analysis. PLoS ONE.

[39] Shoukry N.K., Hanafy A.F., Ashour A.A., Yamani A.S., Elhafez N.M., Elhafez S.M. (2017). Weight-bearing joint position sense in females with pelvic asymmetry. Bull. Fac. Phys. Ther..

[40] Zhou X., Wang Q., Zhang X., Chen Y., Shen H., Jiang Y. (2012). Effect of pelvic obliquity on the orientation of the acetabular component in total hip arthroplasty. J. Arthroplast..

[41] Otayek J., Bizdikian A.J., Yared F., Saad E., Bakouny Z., Massaad A., Assi A. (2020). Influence of spino-pelvic and postural alignment parameters on gait kinematics. Gait Posture.

[42] Neumann D.A. (2019). Kinesiology of the Musculoskeletal System: Foundations for Rehabilitation.

[43] Donati D., Giorgi F., Farì G., Tarallo L., Catani F., Tedeschi R. (2024). The influence of pelvic tilt and femoral torsion on hip biomechanics: Implications for clinical assessment and treatment. Appl. Sci..

[44] Kobayashi N., Higashihira S., Kitayama H., Kamono E., Yukizawa Y., Oishi T., Inaba Y. (2021). Effect of decreasing the anterior pelvic tilt on range of motion in femoroacetabular impingement: A computer-simulation study. Orthop. J. Sports Med..

[45] Ismail K.K., Lewis C.L. (2022). Effect of simulated changes in pelvic tilt on hip joint forces. J. Biomech..

[46] Kuhns B.D., Kahana-Rojkind A.H., Parsa A., McCarroll T.R., Sikligar D., Domb B.G. (2025). Posterior pelvic tilt allows for increased hip motion, while anterior pelvic tilt decreases joint stress: A systematic review of biomechanical and motion analyses. Arthroscopy.

[47] Gu J.S., Choi S.J., Choi H.S., Shin W.S. (2016). Effects of pelvic tilt training using inclinometer on joint position sense and postural alignment in patients with chronic stroke. J. Korean Phys. Ther..

[48] Jung M., Kim N., Lee Y. (2022). Immediate effect of hip hinge exercise stretching on flexibility of lower limb, pelvic tilting angle, proprioception and dynamic balance in individuals with hamstring tightness. Phys. Ther. Rehabil. Sci..

[49] Alkhamis B.A., Reddy R.S., Alahmari K.A., Alshahrani M.S., Koura G.M., Ali O.I., Elrefaey B.H. (2024). Balancing act: Unraveling the link between muscle strength, proprioception, and stability in unilateral hip osteoarthritis. PLoS ONE.

[50] Endo K., Suzuki H., Nishimura H., Tanaka H., Shishido T., Yamamoto K. (2012). Sagittal lumbar and pelvic alignment in the standing and sitting positions. J. Orthop. Sci..

[51] Bagwell J.J., Fukuda T.Y., Powers C.M. (2016). Sagittal plane pelvis motion influences transverse plane motion of the femur: Kinematic coupling at the hip joint. Gait Posture.

[52] Levangie P.K., Norkin C.C. (2011). Joint Structure and Function: A Comprehensive Analysis.

[53] Han J., Waddington G., Adams R., Anson J., Liu Y. (2016). Assessing proprioception: A critical review of methods. J. Sport Health Sci..

[54] In T.S., Jung J.H., Jung K.S., Cho H.Y. (2021). Spinal and pelvic alignment of sitting posture associated with smartphone use in adolescents with low back pain. Int. J. Environ. Res. Public Health.

[55] Fryer G. (2000). Muscle energy concepts: A need for change. J. Osteopath. Med..

